# Expansion of regulatory T cells by CD28 superagonistic antibodies attenuates neurodegeneration in A53T-α-synuclein Parkinson’s disease mice

**DOI:** 10.1186/s12974-022-02685-7

**Published:** 2022-12-31

**Authors:** Mohammad Badr, Rhonda L. McFleder, Jingjing Wu, Susanne Knorr, James B. Koprich, Thomas Hünig, Jonathan M. Brotchie, Jens Volkmann, Manfred B. Lutz, Chi Wang Ip

**Affiliations:** 1 Department of Neurology, University Hospital of Würzburg, Würzburg, Germany; 2 Krembil Research Institute, Toronto Western Hospital, University Health Network, Toronto, ON Canada; 3 Institute for Virology and Immunobiology, University of Würzburg, Würzburg, Germany; 4 Atuka Inc, Toronto, ON Canada

**Keywords:** Parkinson’s disease, Neuroinflammation, T cells, Regulatory T cells, Neuroprotection

## Abstract

**Background:**

Regulatory CD4^+^CD25^+^FoxP3^+^ T cells (Treg) are a subgroup of T lymphocytes involved in maintaining immune balance. Disturbance of Treg number and impaired suppressive function of Treg correlate with Parkinson’s disease severity. Superagonistic anti-CD28 monoclonal antibodies (CD28SA) activate Treg and cause their expansion to create an anti-inflammatory environment.

**Methods:**

Using the AAV1/2-A53T-α-synuclein Parkinson’s disease mouse model that overexpresses the pathogenic human A53T-α-synuclein (hαSyn) variant in dopaminergic neurons of the substantia nigra, we assessed the neuroprotective and disease-modifying efficacy of a single intraperitoneal dose of CD28SA given at an early disease stage.

**Results:**

CD28SA led to Treg expansion 3 days after delivery in hαSyn Parkinson’s disease mice. At this timepoint, an early pro-inflammation was observed in vehicle-treated hαSyn Parkinson’s disease mice with elevated percentages of CD8^+^CD69^+^ T cells in brain and increased levels of interleukin-2 (IL-2) in the cervical lymph nodes and spleen. These immune responses were suppressed in CD28SA-treated hαSyn Parkinson’s disease mice. Early treatment with CD28SA attenuated dopaminergic neurodegeneration in the SN of hαSyn Parkinson’s disease mice accompanied with reduced brain numbers of activated CD4^+^, CD8^+^ T cells and CD11b^+^ microglia observed at the late disease-stage 10 weeks after AAV injection. In contrast, a later treatment 4 weeks after AAV delivery failed to reduce dopaminergic neurodegeneration.

**Conclusions:**

Our data indicate that immune modulation by Treg expansion at a timepoint of overt inflammation is effective for treatment of hαSyn Parkinson’s disease mice and suggest that the concept of early immune therapy could pose a disease-modifying option for Parkinson’s disease patients.

**Supplementary Information:**

The online version contains supplementary material available at 10.1186/s12974-022-02685-7.

## Introduction

Although generally not considered an autoimmune disease, neuroinflammation still plays an essential role in the pathogenesis of Parkinson’s disease (PD). This is evidenced by pro-inflammation observed in the nigrostriatal tract of human PD brain autopsies and in mouse models of PD with activation of microglia, infiltration of T cells, and elevation of pro-inflammatory cytokines [1–6]. Moreover, T cells that specifically recognize PD-associated α-synuclein (αSyn)-derived epitopes, were detected in blood of PD patients [7, 8]. CD4^+^CD25^+^FoxP3^+^ regulatory T cells (Treg) are a subgroup of T cells that are important in maintaining immune homeostasis and self-tolerance due to their immune suppressive capacity on effector T cells (Teff) [9]. They are involved in regulating cellular immunity in pathologic conditions such as autoimmune diseases, cancer and infections [10–12]. Mutations of FoxP3 lead to dysfunction of Treg with development of various autoimmune diseases, inflammatory bowel diseases and allergic reactions in human and mice [13–15]. Activation of Treg and converting microglia to an anti-inflammatory mode, has been demonstrated to be neuroprotective in preclinical models of PD [16–20]. In addition, an upregulation of neurotrophin secretion (brain-derived neurotrophic factor/BDNF), by Treg, has been implicated in mediating neuroprotection in an HIV-1 mouse model [21]. The CD4^+^FoxP3^+^ Treg numbers have been reported to be elevated in sera of PD patients by several groups [22, 23]. However, an impaired ability of Treg to suppress Teff was described in PD patients [24, 25], thereby pointing towards a dysregulated Treg activity in PD promoting pro-inflammation. Moreover, suppressive function of dysfunctional Treg from PD patients was restored after ex vivo expansion [25], thereby rendering Treg expansion to induce anti-inflammation interesting. Indirect means to elicit a beneficial effect via Treg mobilization in preclinical PD models have been attempted, for example, by Bacillus Calmette–Guérin (BCG) vaccination [17, 18, 26], by delivery of a vasoactive intestinal peptide receptor-2 (VIPR2) peptide agonist [27] and by granulocyte–macrophage colony stimulation factor (GM-CSF) administration [28, 29] that even conducted in a phase 1 clinical trial [30, 31]. However, a direct way to activate Treg in a PD mouse model remains to be tested. CD28 superagonists (CD28SA) are monoclonal antibodies directed against the homodimeric CD28 receptor of T cells and are available for the use in mice (D665). CD4^+^CD25^+^ Treg and Teff express the CD28 receptor, which is the key co-stimulatory molecule on T cells. By cross-linking to the CD28 receptor only T cell subgroups become activated that recently experienced either a weak tonic T cell receptor signal generated by MHC scanning, or a stronger signal as a result of cognate antigen recognition as is the case for Treg cells which are continuously stimulated by self-antigens. Employing low CD28SA doses therefore allows to selectively address Treg cells in both mice and humans [32, 33].

The target of CD28SA is proposed to be Treg cells in the periphery. In animal models of multiple sclerosis and stroke, CD28SA-triggered Treg were shown to suppress inflammation in the periphery and to migrate to the CNS where they exert their effect by turning the environment from pro-inflammatory to anti-inflammatory and neuroprotective [34, 35]. By this mode of action, we aimed to alter the disease course in a pathogenetically relevant mouse model of PD that demonstrates an overexpression of human mutated A53T-αSyn in dopaminergic neurons of the substantia nigra (SN) after injection of an A53T-αSyn encoding viral vector (AAV1/2) [36]. AAV1/2-A53T-αSyn (hαSyn) mice exhibit a high face (insoluble Lewy-like pathology and motor deficits) and construct (model based on genetics of PD) validity coupled with neuroinflammation [6] and are thereby optimally suited to investigate the neuroprotective and anti-inflammatory efficacy of CD28SA.

## Material and methods

### Animals and stereotaxic surgery

Male wildtype C57BL/6 mice from Charles River Laboratories, Sulzfeld, Germany, were kept at near pathogen-free environment under standard conditions (21 °C, 12 h/12 h light/dark cycle). At the age of 13 weeks, a total of 236 mice (176 main experiment, 40 adoptive transfer experiment, 20 staggered start trial) were unilaterally injected in the right SN (Bregma: AP − 3.1 mm; ML − 1.4 mm; DV − 4.2 mm) with 2 µl of hαSyn or Empty Vector AAV1/2 (EV) as described [6]. AAV1/2 solutions were infused by a microinjector (Stoelting Co., Wisconsin, USA) at a rate of 0.2 µl/min using a 75 N 5 µl Hamilton Syringe at a concentration of 5.16 × 10^12^ genomic particles (gp)/ml. Genedetect^®^ provided AAV1/2 and controlled equal number of genomic particles in EV and hαSyn AAV1/2. General anesthesia was applied in all surgical procedures using isoflurane.

### In vivo delivery of CD28SA

**Fig. 1:**
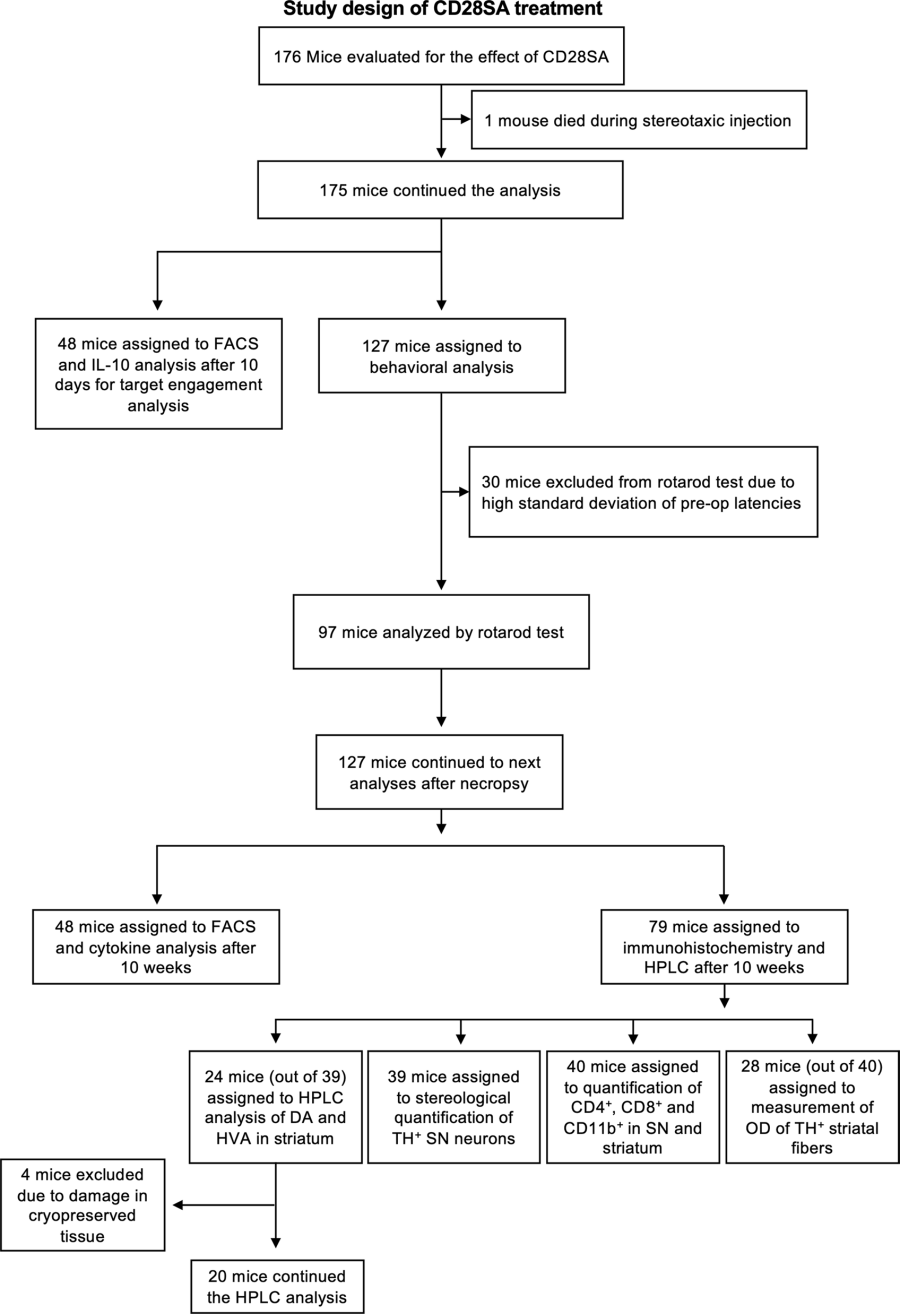
Study design for the CD28SA treatment in hαSyn PD mice

**Fig. 2:**
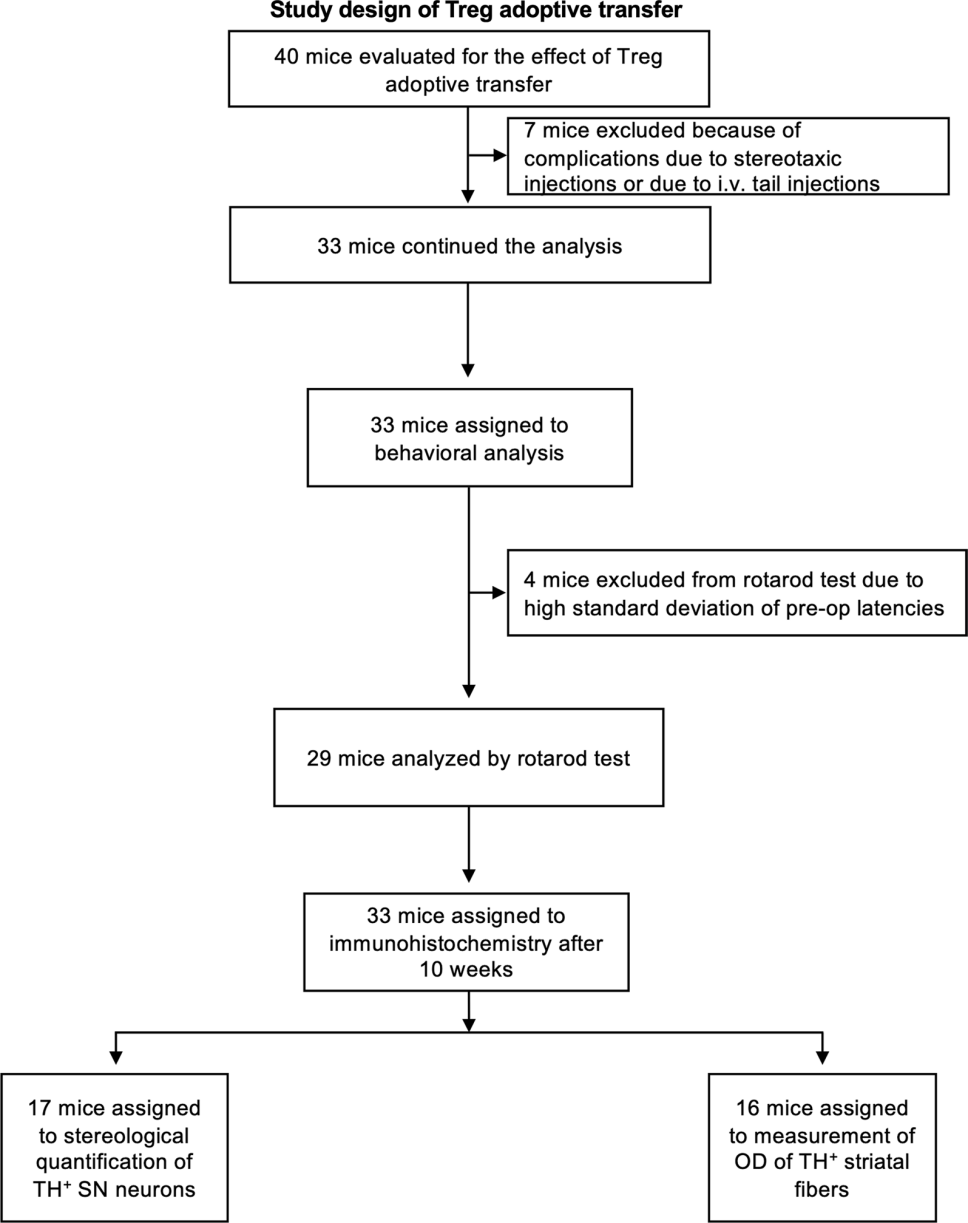
Study design for the adoptive transfer of Treg in hαSyn PD mice

Mice received intraperitoneal (i.p.) injections of either 200 µl of mouse anti-mouse CD28SA (D665) in PBS solution at a concentration of 1 mg/ml (InVivo BioTech Services, Hennigsdorf, Germany) or 200 µl of 0.1 M sterile PBS. The study designs of the main study and the adoptive transfer study are presented in Figs. 1 and 2, respectively.

### Adoptive transfer of Treg

#### Treg sorting and isolation

Treg were isolated from 10 donor mice whose Treg population was previously expanded by i.p. injections of CD28SA. Treg isolation was done using CD4^+^CD25^+^ selection kit from Miltenyi Biotec and magnetic separation was done using MS columns. Spleen and lymph nodes of 10 Treg-enriched donor mice were processed by forcing cells through 70-µm cell strainers. Red blood cells in spleens were lysed by 2-min incubation in an NH^4^Cl-based RBS lysis buffer. The total count of harvested white blood cells from all mice was 9.92 × 10^8^ cells. Harvested cells were labeled with a cocktail of antibodies against CD8, CD14, CD15, CD16, CD19, CD36, CD56, CD123, TCRg/d and CD235 and the labeled cells were depleted over a MACS column. The flow-through cell suspension contained negatively selected CD4^+^ T cells with a count of 3.57 × 10^8^ cells. Pre-enriched CD4^+^ T cells were labeled with CD25 Microbeads in order to positively select CD4^+^CD25^+^ Treg. Count of isolated Treg was 95.3 × 10^6^ cells. A sample of Treg was analyzed by FACS to check for purity.

#### Treg adoptive transfer

Isolated Treg were injected at 2 × 10^7^ cells intravenously in a volume of 350 μl into recipient mice that received hαSyn stereotaxic injection 1 week earlier. Blood samples of recipient mice were drawn from facial vein to check the percentage of CD4^+^CD25^+^ Tregs by FACS analysis, compared to 22 mice that received only a PBS i.v. injection as sham control.

### Behavioral tests

#### Accelerating rotarod

Motor performance of mice was analyzed by the rotarod test (RotaRod Advanced, TSE systems) with an accelerating speed from 5 to 50 rpm for a duration of 300 s. Latency to fall was recorded. Pre-operative measurements were performed on the 3rd day after a 2-day training. For each mouse, a total of 5 runs were carried out per measurement and latency time was calculated as the average excluding the highest and lowest values. Data are shown as the percentage of latency time at the 5th and the 9th week relative to pre-operative values.

### Immunohistochemical staining and quantification of dopaminergic neurons

10 µm fresh coronal cryosections of the SN and striatum were stained for CD4^+^, CD8^+^, CD11b^+^ and GFAP^+^ profiles. 40 μm PFA-fixed coronal cryosections were processed for unbiased stereology. For measurement of proteinase K-digestions, sections were pretreated with proteinase K (20 μg/mL, Sigma, P2308) for 10 min at 37 °C prior to the blocking step. Complete digestion was verified by lack of TH signal. Following blocking, sections were incubated with either rat anti-mouse CD4^+^ (1:1000, Serotec, cat # MCA1767), rat anti-mouse CD8^+^ (1:500, Serotec, cat # MCA609G), rat anti-mouse CD11b (1:100, Serotec, cat # MCA74G) rabbit anti-mouse GFAP (1:5000, Novus Biologicals, cat # 05198, chicken anti-TH (1:500, Abcam #ab76442), rabbit anti-human αSyn (1:10,000, Sigma #S3062) antibodies followed by biotinylated rabbit anti-rat and goat anti-rabbit secondary antibodies (Vector Labs, cat # BA-4001 and BA-1000) and (DAB)-HCl-peroxidase (Vector Labs) development or fluorescent-coupled antibodies: goat anti-rabbit Cy3 (1:300, Abcam, #ab150175), goat anti-chicken AF647 (1:300, Abcam #ab150175). For unbiased stereology free floating sections were incubated with rabbit anti-mouse TH antibody (1:1000; abcam, Cambridge, UK) overnight at 4 °C, followed by incubation with biotinylated goat anti-rabbit antibodies and avidin–biotin–peroxidase reagent and finally with (DAB)–HCl–peroxidase. T cells and microglia were quantified at a magnification of 200× in the region of the SN and striatum as depicted by consecutive sections stained for TH on a BH2 light microscope (Olympus). Mean fluorescence-signal intensity (MFI) of the GFAP and PK-resistant stainings were measured on 8-bit color images without adjustments. For GFAP signal, average MFI was measured within a minimum of two areas within the SN of mice. For PK-resistant αSyn, the SN pars compacta (SNpc) was first outlined based on the TH signal in undigested slices. This outline was then overlayed onto the PK-digested slices to analyze the PK-resistant signal in this region. Two ROIs were drawn to assess the background, the mean background intensity was then subtracted from the total fluorescence. For GFAP, the results were normalized to EV + PBS control animals. Nigral neurons were quantified by unbiased stereology according to the optical fractionator method using a 100x/1.25 numerical aperture objective on a BX53 microscope (Olympus), and a Stereo Investigator software package (version 11.07; MicroBrightField Biosciences, Williston, VT). 40 µm sections covering the extent of the SN and separated by 200 µm (1/5 series) were used for counting dopaminergic (TH^+^) neurons and total (Nissl^+^) neurons in both pars compacta and pars reticulata regions of the SN. Parameters: grid size 110 × 110 μm, counting frame 50 × 50 μm, 2 μm guard zone. Actual mounted thickness was calculated as an average of the measured thickness of each counting site. A Gundersen coefficient of error (for m = 1) of < 0.1 was accepted.

### Catecholamine quantification by high-performance liquid chromatography (HPLC)

Brain sections were homogenized in 200–750 μl of 0.1 M TCA (10 − 2 M sodium acetate, 10 − 4 M EDTA, 10.5% methanol), centrifuged at 10,000*g* for 20 min, supernatants were collected, and pellets were stored for protein analysis. Catecholamines were evaluated using a specific HPLC assay with an Antec Decade II (oxidation: 0.5) electrochemical detector operated at 33 °C. Supernatant samples were injected by a Water 717 + autosampler onto a Phenomenex Nucleosil (5u, 100A) C18 HPLC column (150 × 4.60 mm). Analytes were eluted with a mobile phase of 89.5% 0.1 M TCA, 10 − 2 M sodium acetate, 10 − 4 M EDTA, 10.5% methanol, followed by delivery of the solvent at 0.8 ml/min with a Waters 515 HPLC pump. Analytes were examined in the following order: 3,4-dihydroxyphenylacetic acid (DOPAC), dopamine (DA), homovanillic acid (HVA). Waters Empower software was used for HPLC control and data acquisition. Total protein for each sample was determined with Pierce BCA protein assay, and the amount of catecholamines was expressed as ng analyte/mg total protein.

### Fluorescence-activated cell sorting (FACS) and cytokine assay

#### Flow cytometry

Cell suspensions from cervical lymph nodes, spleen and brain were incubated with CD4 Alexa Fluor 647, CD8 PerCP/Cy5.5, CD25 Alexa Fluor 488 and CD69 Alexa Fluor 488 (Biolegend) for 30 min, subsequently fixed and permeabilized by eBioscience Fix/Perm buffer for 30 min and then incubated with FoxP3-PE antibody (Biolegend) for 1 h. Acquisition was performed on an LSRII flow cytometer (BD) and data analysis was done using FlowJo software (FLOWJO, LLC). In all FACS measurements, compensation controls have been employed using single stained samples of CD4 Alexa Fluor 647 (APC channel), CD8 PerCP/Cy5.5 (PerCP Channel), CD4 PE (PE channel) or CD4 Alexa Fluor 488 (FITC channel).

#### Cytokine analysis

For cytokine analysis, cell suspensions from brain, lymph nodes and spleen were obtained as described above. 2 × 10^5^ cells from each suspension were plated in a 96-well U-bottom plate and the cells were re-stimulated with PMA (0.1 µg/ml, Sigma-Aldrich), Ionomycin (1 µg/ml, Sigma-Aldrich) for 16 h. Supernatant was collected and stored in − 20 °C until further analysis. Cytokine detection was performed using the LEGENDPlex™ Multi-Analyte Flow Assay Kit from BioLegend and data were acquired on an LSRII flow cytometer (BD).

### Statistics

Normality of each data set was investigated by the Q–Q plots. For analysis of more than two groups in striatal TH^+^ optical density, stereological estimation of SN cell numbers, rotarod tests, HPLC analyses, estimation of lymphocyte numbers, FACS analyses, cytokine analyses, the parametric one-way ANOVA was used, followed by Tukey’s multiple comparison test. For analysis of more than two groups in IL10-measurement in CLN and spleen, quantification of CD8^+^ cells in striatum, IL-2 measurement in brain and FACS analysis of CLN at the 10-week timepoint and IL-2 measurement in spleen at 10-day timepoint, the non-parametric Kruskal–Wallis test was used, followed by Dunn’s test. Unpaired, two-tailed Student’s *t*-test was used for comparing striatal TH^+^ optical density, stereological estimation of SN cell numbers, rotarod tests and estimation of lymphocyte numbers, between two groups. *P < 0.05, **P < 0.01, ***P < 0.001 were considered as significant P-values.

## Results

### CD28SA injection leads to early in vivo expansion of Treg and increase of IL-10 level as sign of successful target engagement in hαSyn PD mice

**Fig. 3:**
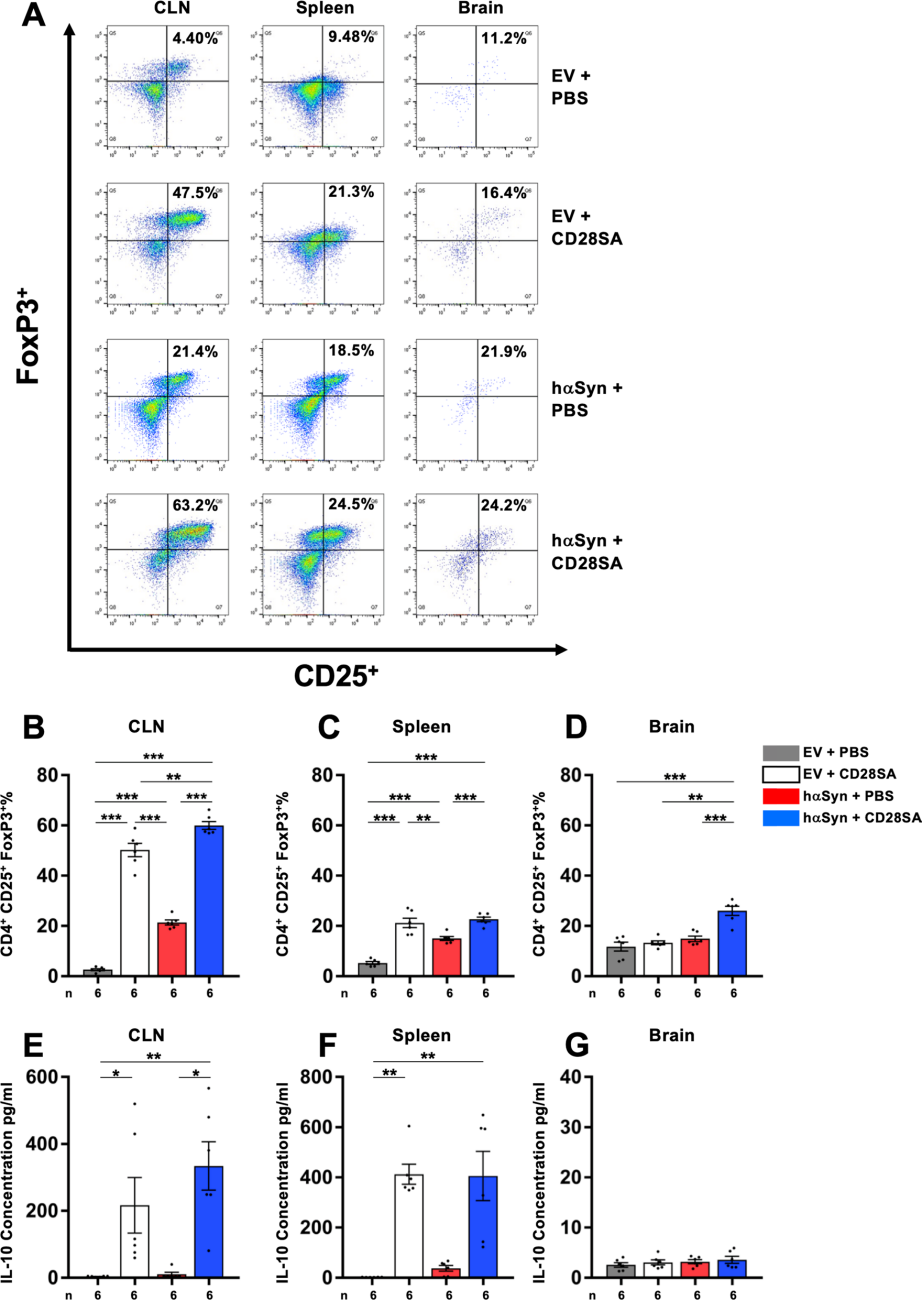
Assessment of target engagement by expansion of Treg after CD28SA injection. **A** Representative FACS analysis images showing the CD4-gated CD25^+^FoxP3^+^ Treg populations in cervical lymph nodes (CLN), spleen and brain of empty vector (EV) or hαSyn vector-injected mice treated either with PBS or with CD28SA, 3 days after respective i.p. injections. **B-D** Treg percentages in CLN (**B**), spleen (**C**) and brain (**D**). **E–G** IL-10 concentrations in CLN (**E**), spleen (**F**) and brain (**G**). Statistical analysis by one-way ANOVA followed by Tukey’s multiple comparisons test, **B-D** CLN, F(3, 20) = 255.7, ***P*** < 0.0001; spleen, F(3, 20) = 46.26, ***P*** < 0.0001; brain, F(3, 20) = 20.22, ***P*** < 0.0001.; **G** brain, F(3, 20) = 0.643, *P* = 0.5963. **E**, **F** Kruskal–Wallis test followed by Dunn’s multiple comparisons: CLN, H = 17.93, *P* = 0.0005; spleen: H = 19.47, P = 0.0002. *P < 0.05, **P < 0.01, ***P < 0.001. All data are shown as mean ± SEM. *n* = number of biologically independent samples, each sample consisting of pools of two animals

To assess target engagement of CD28SA treatment in hαSyn PD mice, we analyzed in vivo Treg expansion and IL-10 production after CD28SA delivery. 200 µg CD28SA was administered intraperitoneally (i.p.), 7 days after AAV injection to avert potential interaction with AAV infection and transduction and to ensure a reestablished blood–brain barrier integrity after stereotactic brain surgery. CD4^+^CD25^+^Foxp3^+^ Treg expansion and IL-10 levels were assessed 3 days after CD28SA delivery (10 days after AAV injection) by FACS and cytokine analyses, respectively (Fig. 3). PBS-treated hαSyn mice that were used as controls demonstrated an increase of Treg in cervical lymph nodes and spleen compared to EV-PBS controls, thereby indicating peripheral Treg expansion and dysregulation induced by hαSyn itself that was independent from CD28SA. Cervical lymph nodes and spleen of hαSyn PD and EV mice that received CD28SA showed a significant elevation of CD4^+^CD25^+^FoxP3^+^ Treg over both PBS-treated control groups (Fig. 3A–C), thus demonstrating a general expansion of Treg in peripheral lymphatic organs. In contrast, analysis of brain tissue after CD28SA treatment revealed an expansion of Treg in hαSyn PD mice only but not in EV controls (Fig. 3A, D), indicative for a specific infiltration of Treg in the inflammatory brain tissue of hαSyn PD but not EV mice. As a marker for Treg activity the IL-10 levels reflected Treg expansion after CD28SA injection in the peripheral lymphatic organs with elevation in both hαSyn PD and EV cervical lymph nodes and spleen (Fig. 3E, F), while brain IL-10 stayed on low and unaltered levels independent from AAV injection or CD28SA treatment (Fig. 3G). These data indicate a successful target engagement on Treg and brain infiltration in hαSyn PD mice by CD28SA administration.

### Early treatment with CD28SA attenuates motor deficits, ameliorates SN dopaminergic cell death and rescues dopaminergic terminals in the striatum of hαSyn PD mice

**Fig. 4:**
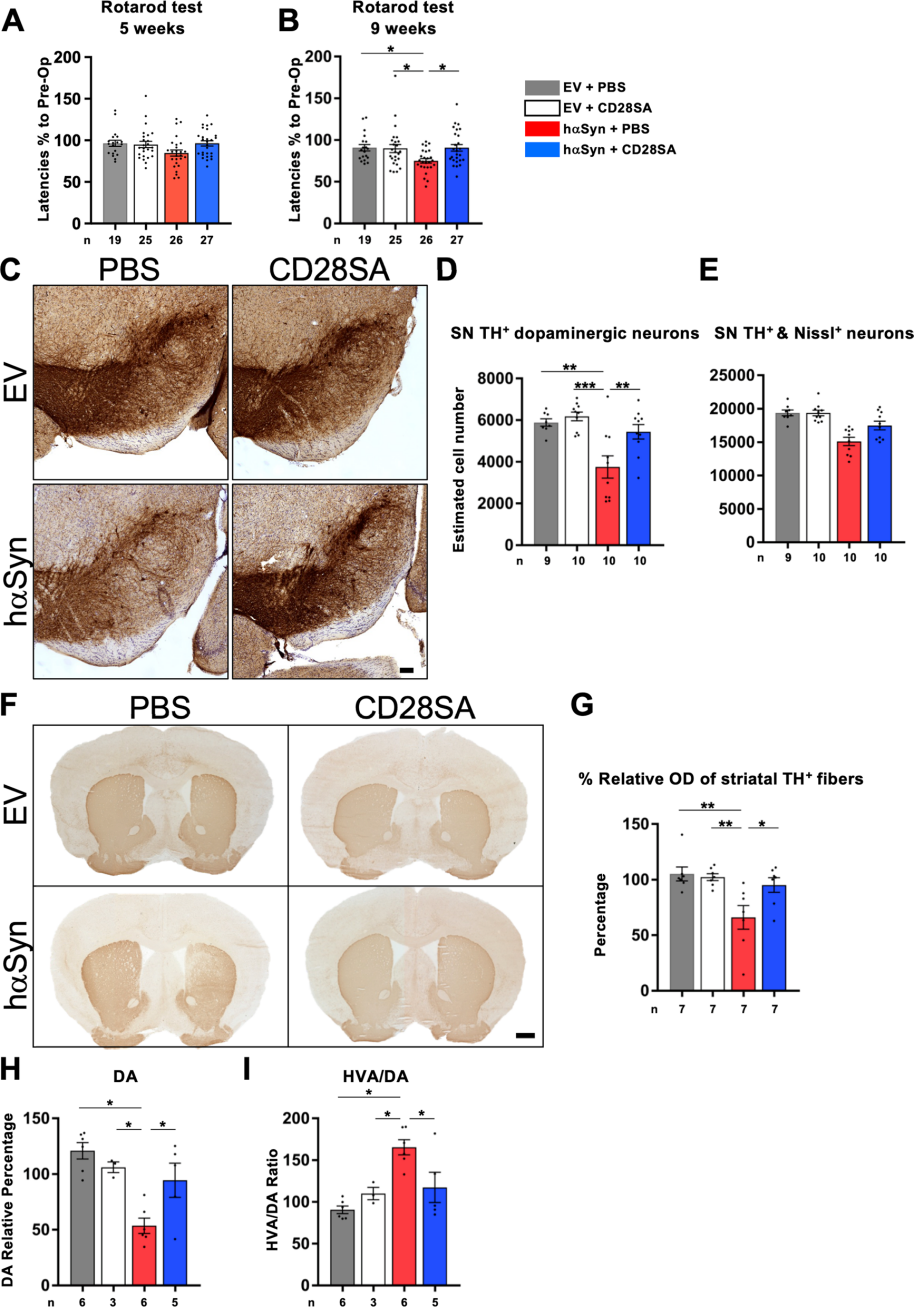
CD28SA ameliorates nigrostriatal dopaminergic degeneration in the hαSyn PD mouse model. **A**, **B** Evaluation of behavioral analyses using the rotarod performance test of EV- or hαSyn-injected mice with either PBS or with CD28SA treatment at five and nine weeks’ timepoint. **C-E** Representative images of TH and Nissl-stained neurons in the SN of the indicated groups of mice (**C**) and estimated cell number by unbiased stereology for TH^+^ dopaminergic SN neurons (**D**) and Nissl^+^ total SN neurons (**E**). **F**, **G** Representative images from the indicated groups of mice showing the striatum after TH^+^ immunostaining (**F**). Results from relative optical density (OD) measurements are shown (**G**). **H**, **I** Analyses of striatal dopamine (DA) levels (**H**) and homovanilic acid (HVA)/DA ratio (**I**). Statistical analysis by one-way ANOVA followed by Tukey’s multiple comparisons test for: Rotarod, F(3, 92) = 3.921, *P* = 0.0110; TH^+^, F(3, 34) = 9.209, *P* = 0.0001; Nissl^+^, F(3, 34) = 13.77, *P* < 0.0001; striatum OD, F(3, 24) = 6.195, *P* = 0.0029; DA, F(3, 16) = 9.805, *P* = 0.0007; HVA/DA, F(3, 16) = 9.361, P = 0.0008. *P < 0.05, **P < 0.01, ***P < 0.001. All data are shown as mean ± SEM. *n* = number of biologically independent animals. Scale bars: **C** 100 µm, **F** 500 µm

Having shown target engagement after CD28SA delivery in hαSyn PD mice, we next assessed the efficacy of a single dose of CD28SA (delivered at 1 week after AAV injection) in alleviating motor deficits and reduction of dopaminergic neurodegeneration. While both EV injected mouse groups (CD28SA and PBS vehicle) showed a CD28SA-independent, unimpaired motor performance in the rotarod performance test, vehicle-treated hαSyn PD mice developed significant deterioration of motor performance 9 weeks, but not yet at 5 weeks, after AAV injection by showing a reduced latency to fall from the rotarod (Fig. 4A, B). CD28SA treatment prevented this deterioration in hαSyn PD mice (Fig. 4A, B). Quantification of dopaminergic SN neurons 10 weeks after disease induction revealed an increased cell loss in PBS-treated hαSyn PD mice compared to both EV groups (Fig. 4C, D). In contrast, CD28SA injection prevented the loss of dopaminergic and total Nissl^+^ neurons in the SN of hαSyn PD mice (Fig. 4C–E). In line with this finding, optical density measurement of TH^+^ fibers demonstrated a rescue of dopaminergic terminals in hαSyn PD mice by CD28SA administration (Fig. 4F, G). In addition, reduction of striatal dopamine levels and pathologically increased dopamine turnover (HVA/DA ratio) in hαSyn PD mice did not occur after CD28SA treatment (Fig. 4H, I). Of note, CD28SA-treatment did not change the insoluble, proteinase K-resistant α-synuclein load in hαSyn PD mice significantly (Additional file 1: Fig. S1). These data demonstrate that CD28SA administration at an early timepoint during disease course rescues nigrostriatal degeneration and reduces motor deficits in hαSyn PD mice.

### Neuroinflammation is reduced in the nigrostriatal tract of CD28SA-treated hαSyn PD mice

**Fig. 5:**
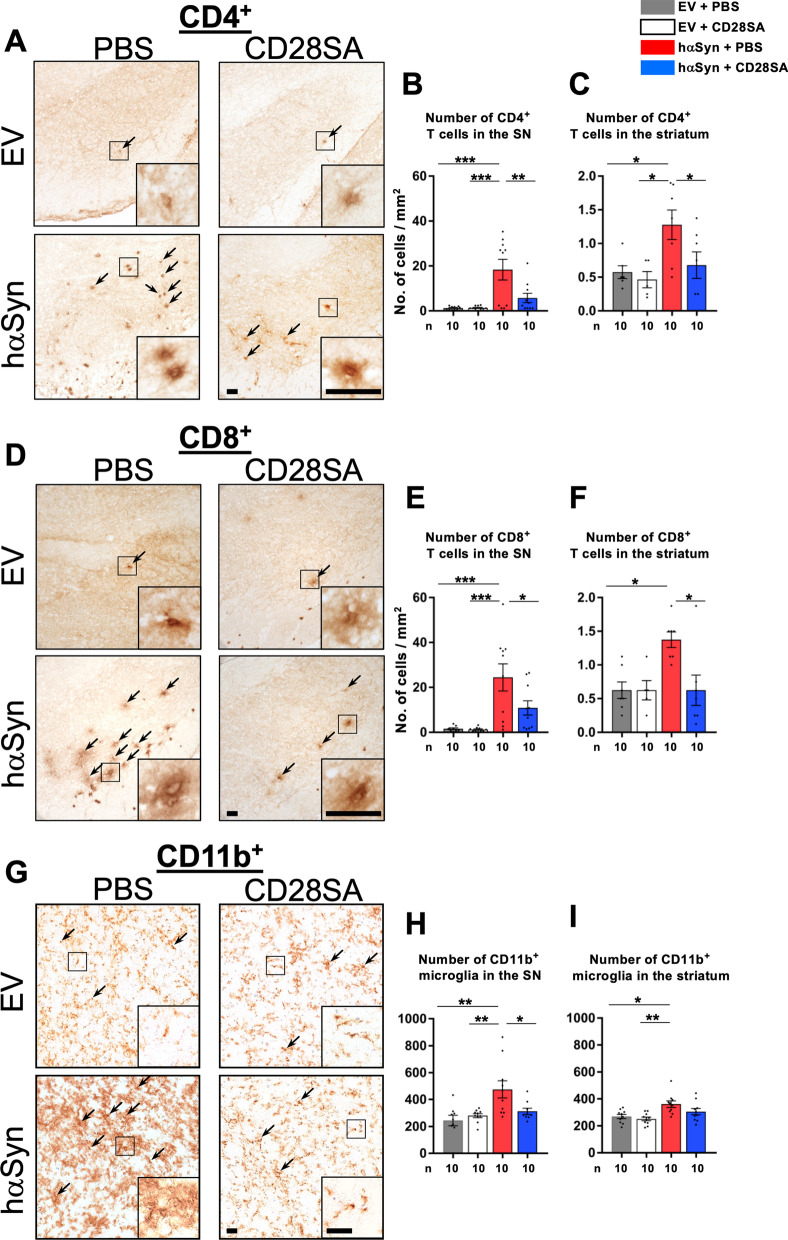
CD28SA reduces neuroinflammation in the nigrostriatal tract of hαSyn PD mice. **A-F** Immunohistochemical stainings and quantification for CD4^+^ (**A–C**) and CD8^+^ (**D–F**) T cells in the SN (**A**, **B**, **D**, **E**) and striatum (**C**, **F**) of EV- or hαSyn-injected mice with either PBS or with CD28SA treatment at 10 weeks’ timepoint. **G-I** Representative images (**G**) and number of CD11b^+^ microglia in the SN (**H**) and the striatum (**I**). Statistical analysis by one-way ANOVA followed by Tukey’s multiple comparisons test: SN CD4^+^, F(3, 36) = 10.16, *P* < 0.0001; SN CD8^+^, F(3, 35) = 9.74, *P* < 0.0001; striatum CD4^+^, F(3, 21) = 4.263, *P* = 0.0168; SN CD11b^+^, F(3, 34) = 6.461, P = 0.0014; striatum CD11b^+^, F (3, 36), *P* = 0.0019. Striatum CD8^+^ count analyzed by Kruskal–Wallis test followed by Dunn’s multiple comparisons test, H = 10.56, *P* = 0.0144. *P < 0.05, **P < 0.01, ***P < 0.001. All data are shown as mean ± SEM. *n* = number of biologically independent animals. Scale bars: 20 µm each

**Fig. 6:**
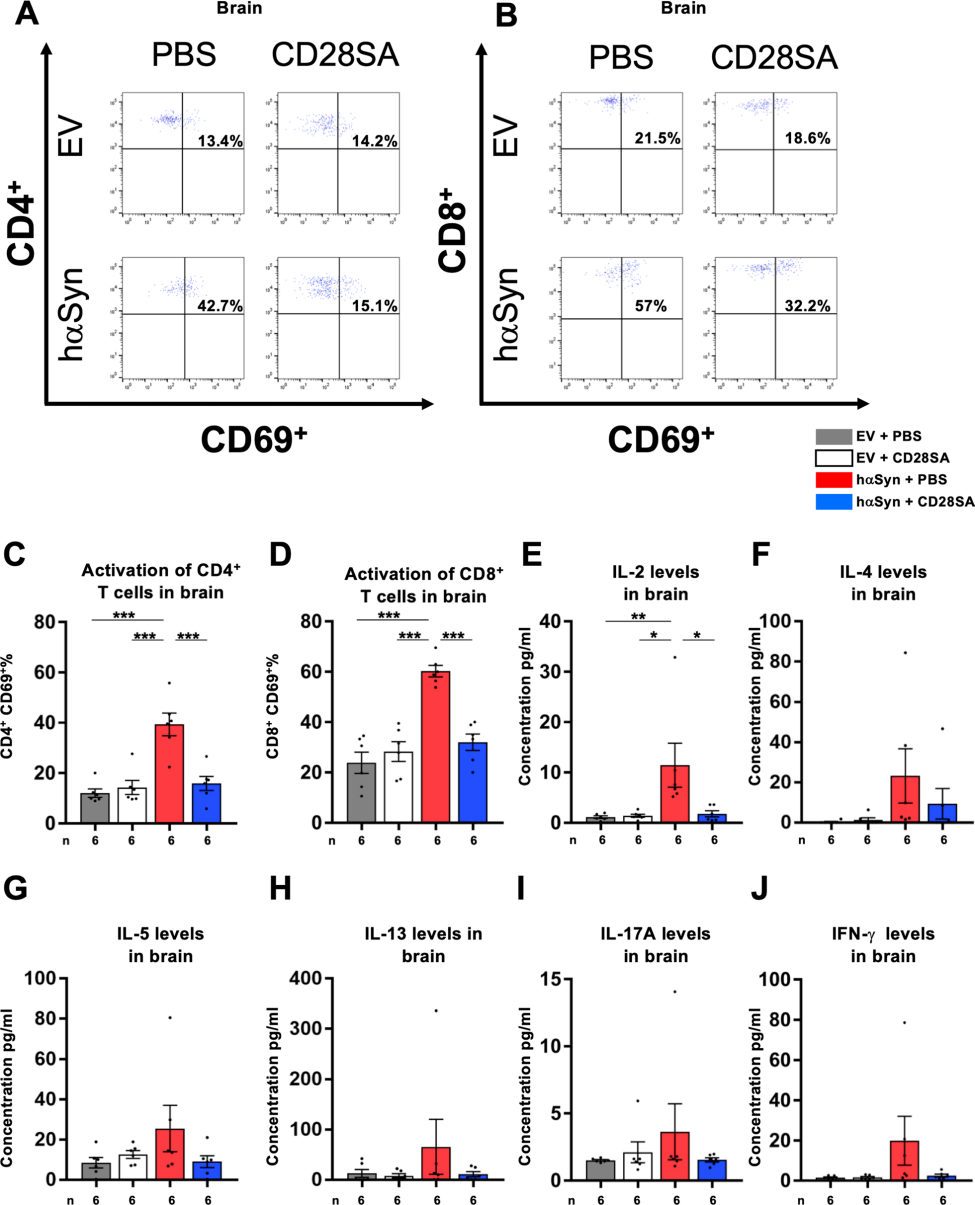
CD28SA decreases activation state of brain T cells in hαSyn PD mice. **A**, **B** Representative FACS analysis images showing the proportion of CD4^+^CD69^+^ (**A**) and CD8^+^CD69^+^ (**B**) activated T cells in the brain of EV- or hαSyn-injected mice treated either with PBS or with CD28SA. **C**, **D** Percentages of CD4^+^CD69^+^ (**C**) and CD8^+^CD69^+^ (**D**) in the brain of the indicated groups as analyzed by FACS. **E-J** Cytokine concentrations in the brain: IL-2 (**E**), IL-4 (**F**), IL-5 (**G**), IL-13 (**H**), IL-17A (**I**) and IFN-γ (**J**). Statistical analysis by one-way ANOVA followed by Tukey’s multiple comparisons test: CD4^+^CD69^+^, F(3, 20) = 16.82, *P* < 0.0001; CD8^+^CD69^+^, F(3, 20) = 22.16, *P* < 0.0001. Statistical analysis by Kruskal–Wallis test followed by Dunn’s multiple comparisons test: IL-2, H = 13.16, *P* = 0.0043. *P < 0.05, **P < 0.01, ***P < 0.001. All data are shown as mean ± SEM. n = number of biologically independent samples, each sample consisting of pools of two animals

To examine the mechanism of action of CD28SA-induced neuroprotection in hαSyn PD mice, we inspected mouse brains 10 weeks after AAV injection with focus on the immune system. In comparison to EV mice, hαSyn PD PBS controls showed significantly elevated CD4^+^ (Fig. 5A–C) and CD8^+^ T cell numbers (Fig. 5D–F) in the SN and the striatum accompanied by increased numbers of CD11b^+^ microglia and other myeloid cell counts (Fig. 5G–I), indicative for neuroinflammation in the hαSyn PD model. In contrast, analysis of GFAP^+^ astrocytes did not show any significant changes in PBS-treated hαSyn PD mice compared to EV controls (Additional file 2: Fig. S2). hαSyn PD mice that had received CD28SA treatment showed significantly reduced numbers of CD4^+^, CD8^+^ T cells and CD11b^+^ myeloid cells (Fig. 5A–I). This finding was underlined by reduced activation of CD4^+^ and CD8^+^ T cells, assessed by CD69 co-staining, in CD28SA-treated hαSyn PD mice compared to PBS vehicle-injected hαSyn PD controls to comparable levels as found in both EV groups (Fig. 6A–D). Moreover, elevation of pro-inflammatory T cell cytokine levels in the brains of PBS-treated hαSyn PD mice compared to EV controls such as IL-2, IL-4, IL-5, IL-13, IL-17, and IFN-γ were decreased after CD28SA treatment (Fig. 6E–J). However, with the exception of IL-2, the differences did not reach statistical significance. Nonetheless, these data show the long-acting anti-inflammatory effect following a single administration of CD28SA.

### Neuroprotective effect of CD28SA in hαSyn PD mice is mediated by Treg

**Fig. 7:**
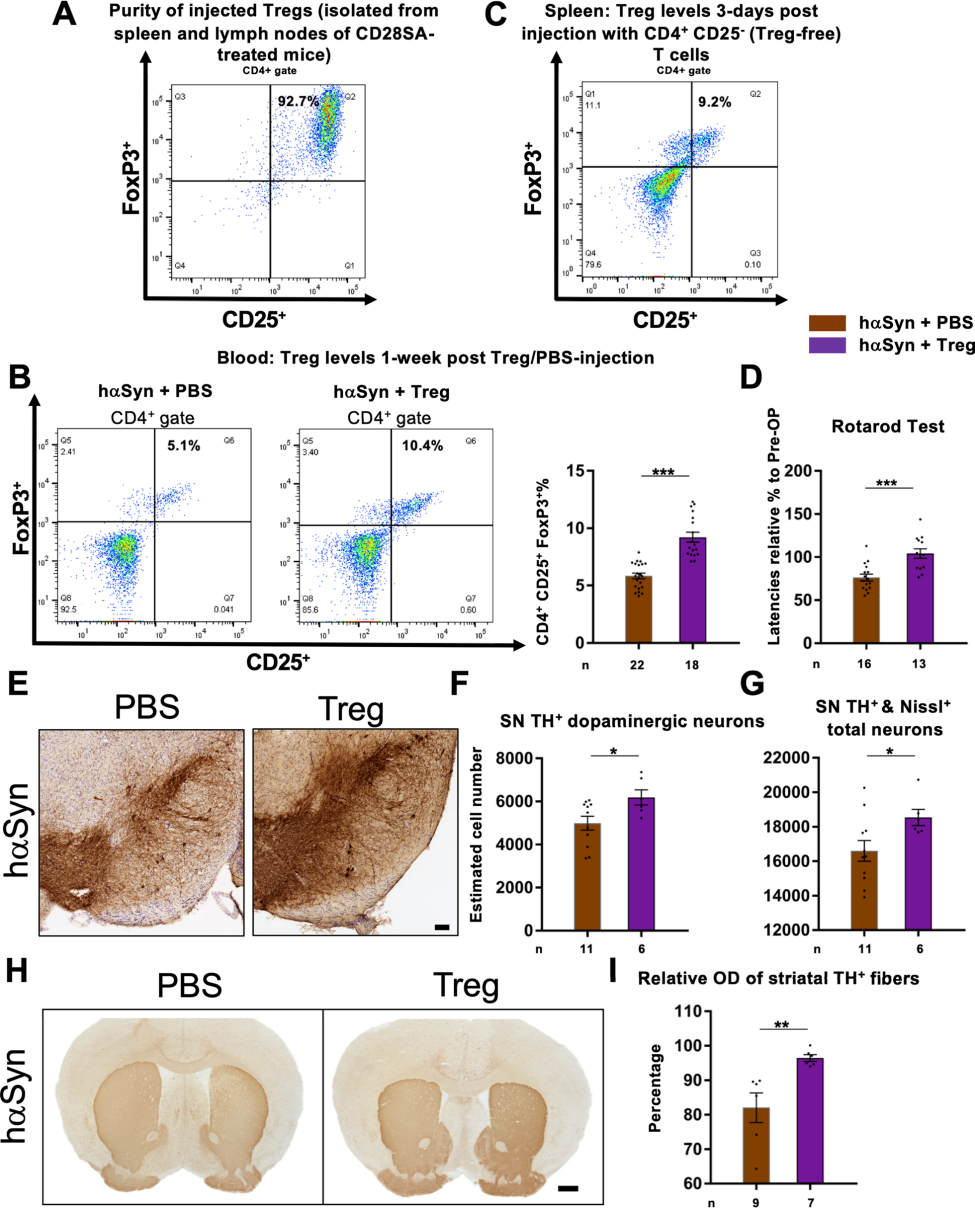
Adoptively transferred Treg reduce dopaminergic degeneration in the nigrostriatal tract of hαSyn PD mice. **A** Representative FACS image of the Treg population in purified cells. **B** Comparison of Treg percentages in mice blood, 1 week after either PBS (control) or Treg injection of hαSyn-injected mice, showing representative images of the FACS analysis as well as statistical comparison. **C** FACS image showing Treg proportion in mouse spleen three days after i.v. injection with a Treg-devoid CD4 + CD25- T cell suspension. **D** Evaluation of behavioral analysis using rotarod performance in the indicated groups of mice. **E–G** Representative images of TH^+^/Nissl^+^ dopaminergic neurons in the SN of the indicated groups of mice (**D**) and estimated cell number by unbiased stereology for TH^+^ SN neurons (**E**) and Nissl^+^ SN neurons (**F**). **H** Representative images from the indicated groups of mice showing the striatum after TH^+^ immunostaining. **I** Results from optical density measurements in TH immunostained striatum. Statistical analysis by unpaired two-tailed *t*-test: blood Treg: t(38) = 7.201, *P* < 0.0001; rotarod: t(27) = 4.204, *P* = 0.0003; TH^+^: t(15) = 2.378, *P* = 0.0311; Nissl^+^: t(15) = 2.171, *P* = 0.0464; striatum OD:: t(10) = 3.279, *P* = 0.0083. *P < 0.05, **P < 0.01, ***P < 0.001. All data are shown as mean ± SEM. n = number of biologically independent animals. Scale bars: **E** 100 µm, **H** 500 µm

To clarify if indeed Treg are capable of mediating neuroprotection in hαSyn PD mice, we adoptively transferred purified Treg into hαSyn PD mice in a proof-of-concept experiment. For this, CD4^+^CD25^+^FoxP3^+^ Treg of wildtype (wt) mice were harvested from lymph nodes and spleen 3 days after CD28SA injection and injected via the tail vein into hαSyn PD mice 1 week after AAV delivery into the SN. Purity of Treg was 93% (Fig. 7A). 1 week after Treg transfer, FACS analyses of peripheral blood revealed an enrichment of Treg in hαSyn PD mice (10.4% of total CD4^+^ T cells) compared to hαSyn PD controls that received i.v. PBS injection (5.1%) (Fig. 7B). To rule out that the observed elevated Treg number after adoptive transfer was due to a direct CD28SA effect, another cohort of mice received CD4^+^CD25^−^ Treg-devoid T cells derived from the same Treg purification process as for the described adoptive Treg transfer experiment. Splenic Treg number of these mice was low (9.2%) (Fig. 7C) clearly contrasting Treg expansion of 47.5–63.2% after CD28SA treatment in hαSyn PD and EV mice (Fig. 3A).

**Fig. 8:**
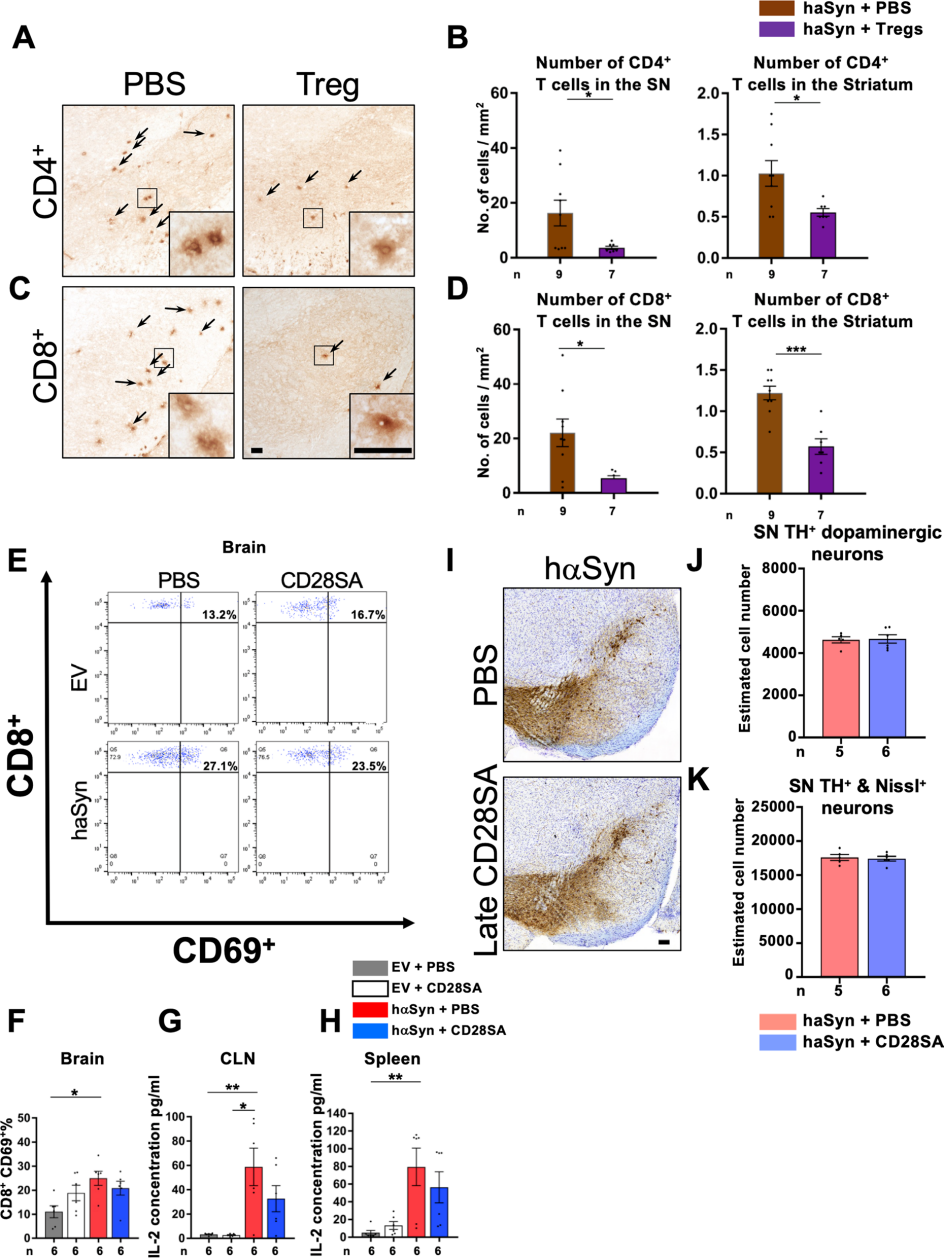
Adoptive transfer of Treg diminishes neuroinflammation in the nigrostriatal tract of hαSyn PD mice; CD28SA decreases early T cell activation in brain and modulates early IL-2 response in peripheral lymphatic organs of hαSyn PD mice; delayed treatment with CD28SA fails to reduce SN dopaminergic neurodegeneration in hαSyn PD mice. **A-D** Immunohistochemical stainings and quantification for CD4^+^ (**A**, **B**) and CD8^+^ (**C**, **D**) T cells in the SN and striatum of hαSyn-injected mice with either PBS or Treg delivery. Statistical analysis by unpaired two-tailed *t*-test: SN CD4^+^: t(14) = 2.367, *P* = 0.0329; striatum CD4^+^: t(14) = 2.601, *P* = 0.0209; SN CD8^+^: t(14) = 2.835, *P* = 0.0132; striatum CD8^+^: t(14) = 5.2, *P* = 0.0001. *P < 0.05, **P < 0.01, ***P < 0.001. Scale bars: 20 µm each. **E** Representative FACS analysis images showing the percentages of CD8^+^CD69^+^ T cells in brain of EV- or hαSyn-injected mice with either PBS or with CD28SA treatment, ten days after AAV injection (3 days after PBS or CD28SA delivery). **F** Percentages of CD8^+^CD69^+^ in brain as analyzed by FACS. **G**, **H** Concentrations of IL-2 in CLN (**G**) and spleen (**H**) of EV- or hαSyn-injected mice treated either with PBS or with CD28SA. Statistical analysis by one-way ANOVA followed by Tukey’s multiple comparisons test: CD8^+^CD69^+^ brain, F(3, 20) = 4.073, *P* = 0.0207); IL-2 CLN, F(3, 20) = 8.186, *P* = 0.0009; statistical analysis by Kruskal–Wallis test followed by Dunn’s multiple comparisons test: IL-2 spleen, H = 12.7, *P* = 0.0053. *P < 0.05, **P < 0.01. Data are shown as mean ± SEM. *n* = number of biologically independent samples, each sample consisting of pools of two animals. **I-K**, Representative images of TH and Nissl-stained neurons in the SN of the indicated groups of mice (**I**) and estimated cell number by unbiased stereology for TH^+^ dopaminergic SN neurons (**J**) and Nissl^+^ total SN neurons (**K**). Statistical analysis by unpaired two-tailed *t*-test. All data are shown as mean ± SEM. *n* = number of biologically independent animals. Scale bar: 50 µm

Ten weeks after AAV delivery, Treg-enriched hαSyn PD mice displayed significantly less motor deficits (rotarod performance test), higher numbers of dopaminergic and total neurons in the SN (Fig. 7D–G) and increased dopaminergic striatal fibers (Fig. 7H, I) compared to PBS-injected vehicle-control hαSyn PD mice. In addition, adoptive Treg transfer reduced the number of CD4^+^ and CD8^+^ T cells in the SN and the striatum of hαSyn mice compared to hαSyn PBS controls (Fig. 8A–D). These data indicate that Treg mediate protection of the nigrostriatal tract and reduction of neuroinflammation in hαSyn PD mice.

### Treg expansion mediates early suppression of inflammation in hαSyn PD mice

Next, we addressed the early impact of CD28SA treatment on the immune system in hαSyn PD mice. For this, we focused on CD8^+^CD69^+^ T cells in the brain and IL-2 levels in the cervical lymph nodes and spleen, since elevation of activated CD8^+^CD69^+^ T cells and IL-2 levels were already described in the respective compartments of hαSyn PD mice at an early stage of 1 week after AAV1/2-A53T-αSyn injection [6]. We observed a significant elevation of activated CD8^+^CD69^+^ T cells in the brain of PBS-treated hαSyn PD mice compared to PBS-treated EV groups 10 days after AAV injection (3 days after CD28SA or PBS delivery) (Fig. 8E, F). In contrast, the increase of CD8^+^CD69^+^ T cells in the brain of hαSyn PD mice after CD28SA treatment was less pronounced and did not differ significantly from EV controls (Fig. 8F). However, the mean reduction by ~ 17% of CD8^+^CD69^+^ cells between CD28SA and PBS-treated hαSyn PD mice did not reach statistical significance. Analysis of IL-2 levels at this early stage of disease, ten days after AAV injection and 3 days after CD28SA or PBS treatment, presented a strong upregulation of IL-2 in PBS-treated hαSyn PD mice in cervical lymph nodes and spleen that was reduced by ~ 30–45% after CD28SA delivery (Fig. 8G, H). These findings indicate an early activation of T cells in hαSyn PD mice that is pronounced in the peripheral immune compartments and is reduced by CD28SA treatment.

### Late treatment with CD28SA fails to reduce SN dopaminergic neurodegeneration in hαSyn PD mice

To address if an early CD28SA treatment is disease-modifying, we examined an additional cohort of hαSyn PD mice, implementing a staggered start trial design. For this, mice were treated with CD28SA 4 weeks after AAV injection at a timepoint where, according to the rotarod data, motor deficits were less pronounced than at the 9-week timepoint. Indeed, this later CD28SA treatment did not prevent degeneration of dopaminergic or total Nissl^+^ SN neurons (Fig. 8I–K). These findings demonstrate that early immune therapy in hαSyn PD mice is neuroprotective over late treatment and point towards a disease-modifying effect of CD28SA.

## Discussion

Treatment strategies for PD are currently aimed at symptomatic relief, whereas causative and disease-modifying therapies are not available to date. Although generally not considered a classical neuroinflammatory disease, evidence is mounting that PD patients suffer from an early shift of the immune system towards pro-inflammation and inflammatory T cell responses contributing to neurodegeneration [6–8, 37]. Dysregulation of CD4^+^ Treg number and activity has been shown to contribute to the development of the pro-inflammatory condition in PD [22–24]. Here we demonstrate in the hαSyn PD mouse model [6, 36] that immune modulation through Treg expansion by CD28SA delivery at an early disease stage prevents the development of a pro-inflammatory profile and reduces dopaminergic neurodegeneration in the nigrostriatal tract of hαSyn PD mice.

Treg are pivotal in suppressing the development of unwanted autoimmune responses and are described to be dysregulated in numbers and function in autoimmune diseases such as rheumatoid arthritis, systemic lupus erythematosus and primary Sjögren’s syndrome among others [38]. In the MPTP mouse model of PD a neuroprotective and anti-inflammatory effect of CD4^+^ Treg has already been shown by either adoptively transferring Treg [16, 19, 39, 40] or indirect Treg expansion [17, 18, 28, 29]. Moreover, in PD patients, safety and immunomodulatory effects of sargramostim, a recombinant human GM-CSF that induces Treg via tolerogenic dendritic cells [41], was demonstrated in a phase 1 trial [30, 31].

Here, we tested if a direct way to expand and activate Treg by use of a single dose of CD28SA given at an early disease stage reduces neurodegeneration in the hαSyn PD mouse model. This model has already been shown to faithfully recapitulate many pathophysiological hallmarks of human PD including progressive motor deficits, dopaminergic neurodegeneration, Lewy-like brain pathology and neuroinflammation [6, 36]. Whether immune modulation can reduce neurodegeneration in this PD model was not demonstrated so far. We found an elevated percentage of CD4^+^CD25^+^FoxP3^+^ Treg among CD4^+^ T cells in peripheral lymphoid organs of hαSyn PD mice already 10 days after disease induction by hαSyn vector injection. This observation indicates a Treg dysregulation in PD mice that is in line with human PD data on disease-related alteration of Treg numbers [22–24]. Of note, the amino acid sequence of the delivered pathologic human A53T-αSyn differs from physiologic mouse αSyn in six amino acids only, thereby putatively acting in parts as a self-antigen and driving the murine self-antigen-specific Treg expansion by the CD28SA to counteract the pro-inflammatory immune response. In line with this observation, fibrillar αSyn was demonstrated to increase the percentage of CD3^+^CD4^+^FoxP3^+^ Treg after subcutaneous inoculation in mice, thereby suggesting that α-synuclein might have a role in controlling Treg generation or expansion [42].

The observed early Treg expansion and elevation of IL-10 levels in cervical lymph nodes and spleen of hαSyn PD and EV mice, 3 days after CD28SA delivery, was in agreement with reports from CD28SA treatment in healthy mice and in models for neuroinflammatory diseases such as glucose-6-phosphate isomerase (G6PI)-induced arthritis as model for rheumatoid arthritis and experimental autoimmune encephalomyelitis (EAE), a model for multiple sclerosis [34, 43, 44]. These data show that Treg of hαSyn PD mice are susceptible to CD28SA treatment despite of the observed dysregulation. In contrast to the pronounced Treg response in peripheral lymphatic organs to CD28SA administration, Treg number in brain was increased only in hαSyn PD mice while CD28SA-treated EV and PBS-injected control groups revealed lower Treg numbers. This is indicative of a migration and a reactivation of Treg in hαSyn PD mouse brains after peripheral priming and activation in cervical lymph nodes by hαSyn proteins that have drained from the brain via lymphatic vessels as previously suggested for this hαSyn PD mouse model [6]. In addition, these data demonstrate that CD28SA acts in the peripheral immune compartment and does not need to penetrate the blood–brain barrier to access the brain to have an impact. Importantly, it was shown in a tumor implantation model that blood–brain barrier integrity is already reestablished 7 days after intracerebral surgery, assessed by ^3^H-mannitol and Evan’s Blue permeability [45], thereby excluding a blood–brain barrier leakage as cause for the increased Treg number in brain of hαSyn PD mice at this early disease stage.

Administration of a single dose of CD28SA at an early disease stage prevented loss of dopaminergic perikarya in the SN and, strikingly, also ameliorated dopaminergic terminal loss in the striatum of hαSyn PD mice over controls that was accompanied by functional motor recovery on the rotarod test. In addition, less neuroinflammation was found in CD28SA-treated hαSyn PD mice compared to PBS controls with decreased numbers of CD4^+^ and CD8^+^ T cells in the nigrostriatal tract, reduced percentage of CD69^+^-activated brain T cells among the CD4^+^ and CD8^+^ population and normalization of elevated IL-2 levels. Interestingly, aggregated α-synuclein pathology, assessed after proteinase K-digestion in hαSyn PD mice did not change significantly after CD28SA-treatment. In line with this finding, in a microglia-specific α-synuclein-overexpression mouse model dopaminergic neurons were observed to degenerate independent of their intraneuronal pathological α-synuclein load [46]. Alleviation of behavioral deficits and attenuation of inflammation by CD28SA-treatment has been observed in various rodent disease models, such as for rheumatoid arthritis and multiple sclerosis and stroke [34, 35, 44]. Moreover, a neuroprotective effect of CD28SA-treatment was shown for models of ischemic stroke resulting in reduction of infarct size [35]. A neuroprotective effect on dopaminergic neurons in the SN by genetic ablation of T cells has been demonstrated in the hαSyn PD mouse model [6]. In addition, experimental deep brain stimulation of the subthalamic nucleus was found to rescue dopaminergic SN perikarya in the equivalent hαSyn PD rat model [47, 48]. However, in both studies dopaminergic terminals and axons still degenerated in the respective hαSyn PD rodents despite the rescue of dopaminergic perikarya. This indicates that CD28SA treatment in hαSyn PD mice has an additional protective effect on axonal degeneration. Of note, a delayed CD28SA therapy at 4 weeks after AAV injection, when ~ 15–20% of SN neurons are already degenerated, did not prevent progressive dopaminergic neurodegeneration, thereby underlining the disease-modifying effect of an early CD28SA treatment. To analyze whether neuroprotection in hαSyn PD mice was mediated by Treg or rather another (direct) CD28SA effect, we adoptively transferred expanded Treg into hαSyn PD mice 1 week after disease induction and observed a rescue of dopaminergic perikarya and terminals compared to hαSyn control mice at 10 weeks. CD4^+^ and CD8^+^ T cell numbers were reduced in SN and striatum of hαSyn PD mice that received Treg. These data underline that the neuroprotective effect of CD28SA-treatment is indeed mediated by Treg. To assess the mechanism of action of Treg expansion in hαSyn PD mice, we analyzed CD8^+^ brain T cell activation with the CD69 marker three days after CD28SA delivery and observed a slight suppression of CD8^+^CD69^+^ T cells in the brain T cells in hαSyn PD mice and, more strikingly, a suppression of early IL-2 expression in cervical lymph nodes and spleen of PD mice of ~ 30–45% three days after CD28SA injection. These data demonstrate that CD28SA has an early anti-inflammatory effect, especially on the peripheral immune compartment of hαSyn PD mice. It is likely that peripheral, CD28SA-expanded Treg control Teff responses directly or indirectly through dendritic cells, leading to reduced Teff activation and infiltration into the CNS and consecutively less microglia activation. This hypothesis is supported by the observation of an early and strong upregulation of IL-2 from T cells in cervical lymph nodes of hαSyn PD mice [6]. As IL-2 is described to promote differentiation of naïve T cells into effector T cells upon antigen-dependent priming [49], hαSyn-specific memory T cells can then infiltrate the brain and become reactivated upon encountering their cognate antigen (α-synuclein-specific peptides) on neurons [50, 51]. Subsequently, activated microglia can then induce neurodegeneration to dopaminergic neurons [46, 52]. In contrast, using the general astrocyte marker GFAP, we did not observe any significant alteration of astrocytes in hαSyn PD mice. In a transgenic hA53T-α-synuclein PD mouse model, activated microglia converted astrocytes to neurotoxic A1 astrocytes [53]. Therefore, future studies are necessary to assess the impact of reactive astrocytes subtypes (A1- and A2-specific) in this hαSyn PD model. Although clinical trials have been undertaken to translate CD28SA into human patients [54], this step remains uncertain. Independent of the method, our data indicate that protocols for Treg expansion in humans may be considered for PD patients.

## Conclusions

Recently, early α-synuclein-specific T cell responses were observed in peripheral blood cells of PD patients and found to precede motor symptoms [8]. Interestingly, T cell reactivity in these patients declined over time. It is therefore attractive to propose that the early, pro-inflammatory disease stage of PD yields a timeframe that is still accessible to immune modulation while at later stages neurodegeneration overtakes inflammation. The concept of early immune therapy for inflammatory neurological diseases has been implemented for multiple sclerosis that is considered a prototypic neuroinflammatory disease. Several clinical studies employing various immune modulators have demonstrated the benefit of early therapy after a first initial neurological attack on long-term clinical outcome [55]. Given the fact that a reduced risk of developing PD by the intake of immunosuppressants was observed just recently in a large population-based case–control study including approximately 48,000 PD patients [56], our data hereby indicate that the concept of early immune therapy could be considered as a disease-modifying option for PD patients.

## Supplementary Information

**Additional file 1: Figure S1.** Pathological aggregation of insoluble α-synuclein in both CD28SA and PBS-treated hαSyn PD mice. **A** Representative immunofluorescence images of PBS or CD28SA-treated hαSyn PD mice SN after α-synuclein and TH immunostaining, with and without proteinase K (± proteinase K). **B** Bar graph depicting the Mean Fluorescent Intensity of proteinase K-resistant αSyn in the SN. Statistical analysis by unpaired two-tailed* t*-test. All data are shown as mean ± SEM. n = number of biologically independent animals. Scale bar: 100 µm

**Additional file 2: Figure S2.** Astrocytes in hαSyn PD mice. **A** Representative images of the SN after GFAP immunostaining. **B** Bar graph depicting the Mean Fluorescent Intensity of GFAP signal in the SN. Statistical analysis by one-way ANOVA followed by Tukey’s multiple comparison test. All data are shown as mean ± SEM. *n* = number of biologically independent animals. Scale bar: 50 µm

## Data Availability

The datasets used and/or analyzed during the current study are available from the corresponding author on reasonable request.
