# Supplementary figures and images for: Expansion of regulatory T cells by CD28 superagonistic antibodies attenuates neurodegeneration in A53T-α-synuclein Parkinson’s disease mice
Source: J Neuroinflammation. 2022 Dec 31;19:319. doi: 10.1186/s12974-022-02685-7 (PMC9805693; doi:10.1186/s12974-022-02685-7)

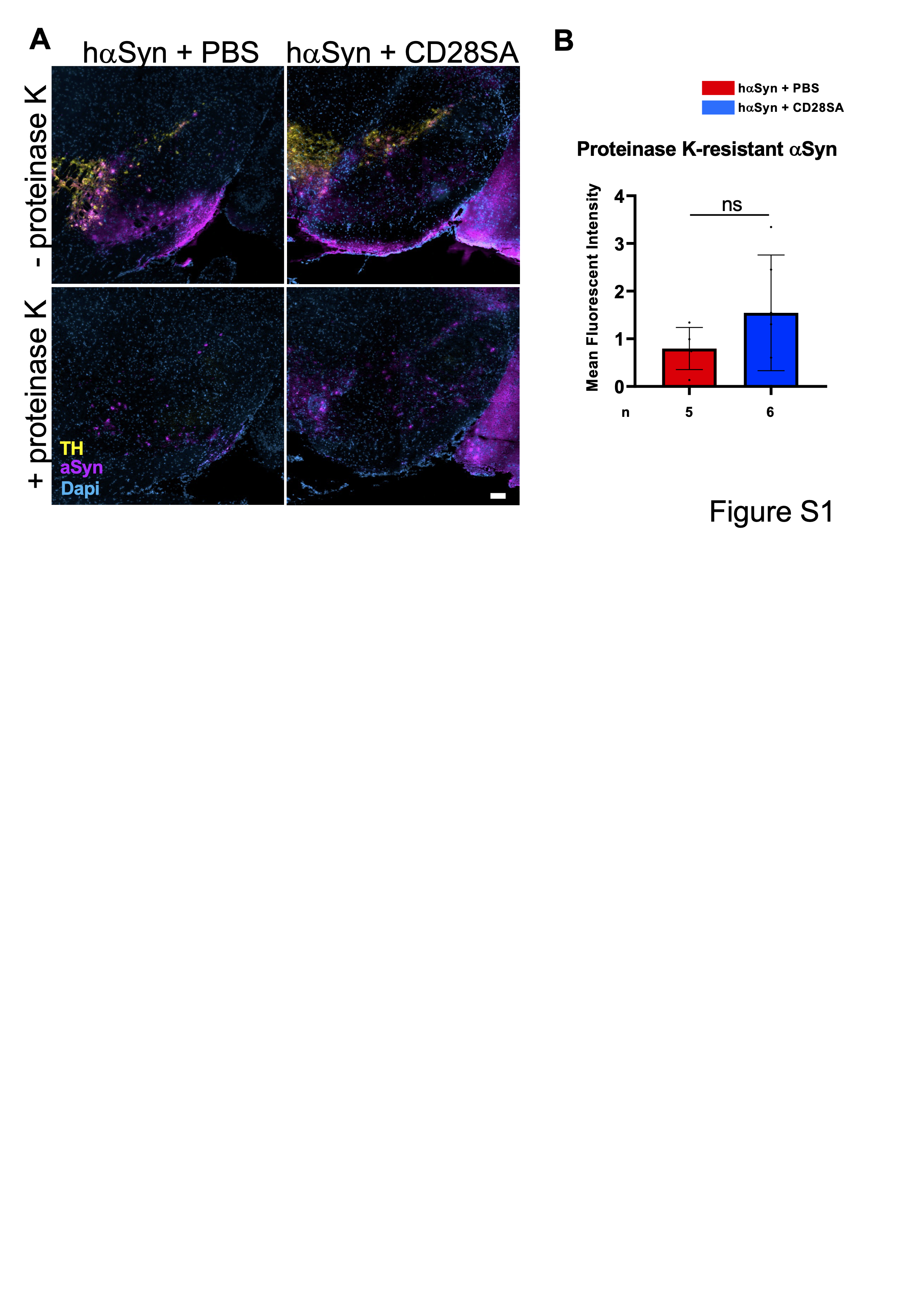

Supplement: Supplementary file 1 — Additional file 1: Figure S1. Pathological aggregation of insoluble α-synuclein in both CD28SA and PBS-treated hαSyn PD mice. A Representative immunofluorescence images of PBS or CD28SA-treated hαSyn PD mice SN after α-synuclein and TH immunostaining, with and without proteinase K (± proteinase K). B Bar graph depicting the Mean Fluorescent Intensity of proteinase K-resistant αSyn in the SN. Statistical analysis by unpaired two-tailed t-test. All data are shown as mean ± SEM. n = number of biologically independent animals. Scale bar: 100 µm [file 12974_2022_2685_MOESM1_ESM.tiff]

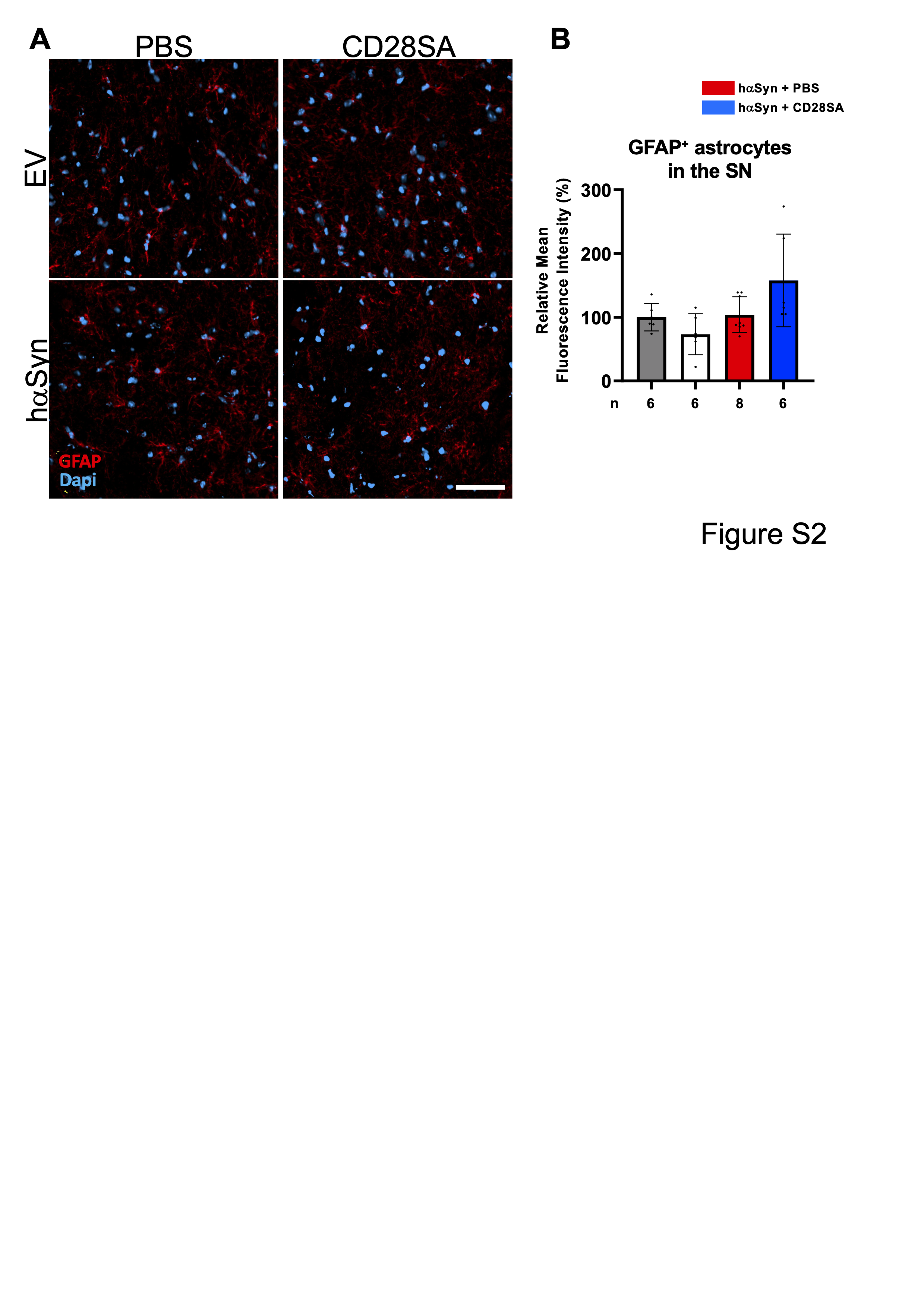

Supplement: Supplementary file 2 — Additional file 2: Figure S2. Astrocytes in hαSyn PD mice. A Representative images of the SN after GFAP immunostaining. B Bar graph depicting the Mean Fluorescent Intensity of GFAP signal in the SN. Statistical analysis by one-way ANOVA followed by Tukey’s multiple comparison test. All data are shown as mean ± SEM. n = number of biologically independent animals. Scale bar: 50 µm [file 12974_2022_2685_MOESM2_ESM.tiff]
